# Impact of Sex on the Therapeutic Efficacy of Rosiglitazone in Modulating White Adipose Tissue Function and Insulin Sensitivity

**DOI:** 10.3390/nu16183063

**Published:** 2024-09-11

**Authors:** Marco Bauzá-Thorbrügge, Emilia Amengual-Cladera, Bel Maria Galmés-Pascual, Andrea Morán-Costoya, Magdalena Gianotti, Adamo Valle, Ana Maria Proenza, Isabel Lladó

**Affiliations:** 1 Grupo de Metabolismo Energético y Nutrición, Departamento de Biología Fundamental y Ciencias de la Salud, IUNICS, Universidad de las Islas Baleares, 07122 Palma, Balearic Islands, Spainemilia.amengual@uib.es (E.A.-C.); andrea.moran@uib.cat (A.M.-C.); magdalena.gianotti@uib.es (M.G.); adamo.valle@uib.es (A.V.); isabel.llado@uib.es (I.L.); 2 Instituto de Investigación Sanitaria Illes Balears (IdISBa), 07120 Palma, Balearic Islands, Spain; 3 Centro de Investigación Biomédica en Red de Fisiopatología de la Obesidad y Nutrición, Instituto de Salud Carlos III, 28029 Madrid, Spain

**Keywords:** white adipose tissue, high-fat diet, rosiglitazone, obesity, inflammation, mitochondria

## Abstract

Obesity and type 2 diabetes mellitus are global public health issues. Although males show higher obesity and insulin resistance prevalence, current treatments often neglect sex-specific differences. White adipose tissue (WAT) is crucial in preventing lipotoxicity and inflammation and has become a key therapeutic target. Rosiglitazone (RSG), a potent PPARγ agonist, promotes healthy WAT growth and mitochondrial function through MitoNEET modulation. Recent RSG-based strategies specifically target white adipocytes, avoiding side effects. Our aim was to investigate whether sex-specific differences in the insulin-sensitizing effects of RSG exist on WAT during obesity and inflammation. We used Wistar rats of both sexes fed a high-fat diet (HFD, 22.5% fat content) for 16 weeks. Two weeks before sacrifice, a group of HFD-fed rats received RSG treatment (4 mg/kg of body weight per day) within the diet. HFD male rats showed greater insulin resistance, inflammation, mitochondrial dysfunction, and dyslipidemia than females. RSG had more pronounced effects in males, significantly improving insulin sensitivity, fat storage, mitochondrial function, and lipid handling in WAT while reducing ectopic fat deposition and enhancing adiponectin signaling in the liver. Our study suggests a significant sexual dimorphism in the anti-diabetic effects of RSG on WAT, correlating with the severity of metabolic dysfunction.

## 1. Introduction

Obesity has become a public health issue worldwide, constituting a silent pandemic and leading to an increase in the incidence of insulin resistance, type 2 diabetes mellitus (T2DM), and cardiovascular diseases (CVD) [1,2]. Unfortunately, while researchers have worked on multiple interventions and treatments, their long-term effectiveness has been limited [3]. For this reason, increasing the available therapeutic options to treat both obesity and its comorbidities is of particular interest.

White adipose tissue (WAT), as the primary lipid reservoir and the largest endocrine organ in the body, plays a pivotal role in managing lipid surplus to prevent peripheral tissue lipotoxicity [4]. Proper adipocyte function relies on adequate mitochondrial activity to handle the excess of fatty acids (FAs), facilitate adipocyte differentiation, and produce adipokines such as adiponectin, which enhances insulin sensitivity and exhibits anti-inflammatory properties. During obesity, the excessive nutrient supply can overwhelm mitochondrial oxidative capacity, leading to the production of reactive oxygen species. This exacerbates the ongoing pro-inflammatory state underlying macrophage infiltration and activation, which is crucial for the development of insulin resistance [5]. Additionally, hypertrophied dysfunctional adipocytes upregulate lipolysis and exhibit an altered secretome, releasing free fatty acids and pro-inflammatory cytokines [6]. These changes trigger systemic lipotoxicity, which could contribute to insulin resistance, thereby predisposing individuals to obesity-related comorbidities such as CVD and T2DM [7]. Consequently, WAT has emerged as an attractive therapeutic target for designing strategies aimed at mitigating the development of insulin-related comorbidities.

Current strategies aimed at preventing and treating obesity and insulin resistance often overlook the impact of sex-specific differences in disease pathways. In fact, sexual differences have been observed both in humans [8] and rodents [9], revealing that diabetes is usually more prevalent in males. This indicates that a distinct therapeutic approach should be used to treat men and women correctly [10]. In this line, previous studies performed in our laboratory involving animal models of dietary obesity revealed a sexual dimorphism in insulin sensitivity and metabolic markers, with female rats exhibiting a better systemic insulin sensitivity profile compared to males. Specifically, females display larger and more functional mitochondria, higher antioxidant activity, enhanced mitochondrial DNA (mtDNA) levels, and reduced oxidative damage across different tissues [11,12,13,14,15,16]. This sex dimorphism has long been attributed to the protective effects of estrogens [17]. In fact, before menopause, women typically exhibit greater insulin sensitivity in skeletal muscle and liver, as well as higher levels of glucose-stimulated insulin secretion, resulting in lower fasting glucose and HbA1c levels compared to men. However, with the onset of menopause, blood pressure and levels of LDL cholesterol (LDL-c) and HbA1c increase, and body fat distribution changes, contributing to the development of glucose intolerance [18]. This dimorphism underscores the importance of tailoring therapeutic strategies to address the particularities of each sex.

Rosiglitazone (RSG), an agonist of peroxisome proliferator-activated receptor gamma (PPARγ), is one of the most potent anti-diabetic drugs within the group of thiazolidinediones (TZD). PPARγ is mainly expressed in WAT and is the major regulator of the proliferation and differentiation of adipocytes, thus becoming one of the main targets of therapeutic strategies against obesity [19]. RSG improves lipid handling by promoting the browning of WAT and the expression of PPARγ target genes [20,21]. As a consequence, the effects of this drug on WAT prevent lipotoxicity, contributing to ameliorating systemic insulin sensitivity in T2DM patients by decreasing serum glucose, insulin and free fatty acid levels. Additionally, RSG can improve insulin sensitivity by specifically targeting the outer mitochondrial membrane protein MitoNEET, which, in turn, induces WAT expansion, mitochondrial activity, and adiponectin secretion, thus reducing obesity-induced inflammation and oxidative stress [22]. 

However, although RSG is considered one of the most potent anti-diabetic drugs, its therapeutic use has been constrained for years by undesired off-target effects associated with the development of CVD and bone fractures, especially in women [23,24]. A recent preclinical study conducted in vitro and in mice has demonstrated a promising strategy for the local delivery of RSG using adipocyte-targeted nanoparticles aimed at achieving adipose tissue-specific effects on key pathways [21]. These innovative strategies promote the function of WAT and hinder the progression of insulin resistance and diabetes without inducing any undesired effects. These findings reignite the possibility of using RSG as an anti-diabetic drug and underscore the importance of conducting further studies aimed at enhancing our understanding of the molecular mechanisms underlying its insulin-sensitizing properties. In sum, given the significant sexual dimorphism in obesity comorbidities and the renewed interest in RSG as an anti-diabetic drug, it is worthwhile to investigate whether there is a sexual dimorphism in the insulin-sensitizing effect of RSG on WAT during obesity and inflammation.

## 2. Material & Methods

### 2.1. Animals and Diets

Six-week-old Wistar rats of both sexes were purchased from Charles River Laboratories (Barcelona, Spain) and kept under controlled conditions of temperature (22 °C), humidity (65 ± 3%) and light (12 h light–dark cycle), with free access to food and water. After a 2-week acclimation period, 8-week-old animals (male and female rats) were placed on a high fat/high sucrose diet (HFD) for 16 weeks. Two weeks before the end of the treatment, male and female rats were randomly divided into two groups with similar mean body weight values, such that one group of males and one group of females received the HFD supplemented with RSG (100 mg/kg of diet, HFD + RSG), which corresponds to a daily intake of 4 mg RSG/kg of body weight. All dietary interventions were sourced from SAFE (Paris, France). HFD was SAFE 235 (4397 kcal/kg; 15.6% proteins, 46% lipids, and 38.5% carbohydrates) with a 28% sucrose content. Food intake was measured weekly, and no differences were observed between groups. To assess insulin sensitivity, animals fed HFD (n *=* 7), and HFD + RSG diets (n *=* 7) were treated with a peritoneal injection of insulin (5 U/kg body weight) 20 min before sacrifice. Non-stimulated animals received an equal volume of saline solution (0.9% NaCl). Female rats were sacrificed at 24 weeks of age in the diestrous phase, as determined by vaginal smears. Immediately after sacrifice, blood was collected to obtain serum by centrifugation at 900× *g* for 10 min at 4 °C. Liver and retroperitoneal WAT were dissected, weighed, frozen in liquid N2, and stored at −80 °C until analysis. White adipocytes from WAT were isolated from fresh tissue using the method described by Rodbell [25]. The average adipocyte size was calculated from the radius measurements of 50 adipocytes individually assessed by microscopy using ImageJ software 1.38e/Java 1.5.0_09 [26,27]. For volume calculation, a spherical model was assumed, using the formula V = 4/3 π r^3^, where V represents the volume and r denotes the radius. Mesenteric and gonadal fat depots were also dissected and weighed to calculate adiposity.

All animal experimental procedures were performed in accordance with the general guidelines approved by our institutional ethics committee (Comité de Bioética de la Universitat de les Illes Balears, COBE No. 3515/2012) and EU regulations (2010/63/UE).

### 2.2. Glucose Tolerance Test and Serum Parameters

A glucose tolerance test was performed 4 days prior to the sacrifice. Rats were fasted overnight, and blood glucose levels were measured from the saphenous vein before and 15, 30, 60, and 120 min after intraperitoneal glucose injection (2 g/kg body weight). Glucose levels were assessed using an Accutrend^®^ GCT meter (Roche Diagnostics, Basel, Switzerland).

The serum levels of insulin (Mercodia, Uppsala, Sweden), FFA and total and HDL cholesterol (HDL-c, Wako Chemicals GmbH, Neuss, Germany), as well as adiponectin and leptin (Millipore, Billerica, MA, USA) were measured using ELISA detection kits. LDL-c was calculated using the Friedewald formula [28].

### 2.3. WAT and Liver Sample Preparation and Analysis

WAT and liver samples were homogenized with a disperser device (IKA T10 basic ULTRA-TURRAX^®^, IKA-Werke GmbH, Staufen, Germany) in STE buffer containing 250 mM sucrose, 20 mM Tris-HCl, 40 mM KCl, and 2 mM EGTA (pH 7.4). WAT homogenates were prepared at 20% *w*/*v* and then centrifuged (600× *g*, 15 min, 4 °C) to remove fat and tissue debris. Liver homogenates were prepared at 10% *w*/*v*. Hepatic PEPCK activity was assessed spectrophotometrically in fresh homogenates, as previously described by Opie et al. [29]. The remaining homogenate volume was stored at −20 °C with phosphatase and protease inhibitors (10 μM leupeptin, 10 μM pepstatin, 1 mM PMSF, and 0.2 mM Na_3_VO_4_) for Western blot analysis. Protein concentrations were measured with the bicinchoninic acid (BCA) protein assay kit (Thermo Fisher Scientific, Waltham, MA, USA) in the WAT and by the Bradford method [30] in the liver.

### 2.4. 3T3-L1 Cell Culture and RSG and Sex Hormones Combined Treatments

Murine 3T3-L1 preadipocytes were purchased at the American Type Culture Collection (Manassas, VA, USA) and were kept at 37 °C in a humidified atmosphere of 5% CO_2_. Cells were grown to confluence in high glucose Dulbecco’s Modified Eagle Medium (DMEM) (Gibco by Invitrogen, Carlsbad, CA, USA), supplemented with 10% newborn calf serum and 1% penicillin-streptomycin (Biological Industries, Beit-Haemek, Israel). Two days after confluence, differentiation was initiated by incubating the cells for 2 days in a differentiating medium containing 10% fetal bovine serum (FBS), 1% penicillin-streptomycin, 0.25 μM dexamethasone, 0.5 mM IBMX, and 5 μM insulin (Sigma-Aldrich, St. Louis, MO, USA). Cells were maintained for 2 additional days in the differentiating medium containing 5 μM insulin. After this period, cells were cultured for 6 more days without insulin, with the culture medium being replaced every 3 days. The cells exhibited a differentiated morphology by day 10, with more than 95% of cells being transformed into mature adipocytes, as evaluated by the detection of lipid droplets by phase contrast microscopy and oil red O staining [31]. The medium was replaced 24 h before treatment with phenol red-free DMEM supplemented with 1% penicillin-streptomycin and 10% charcoal-stripped FBS to avoid estrogenic interference of phenol red and other lipophilic compounds from the serum (Biological Industries, Beit-Haemek, Israel).

On day 11, 3T3-L1 adipocytes were treated with IL-6 (Abcam, Cambridge, UK, ab198572), either alone or in combination with 17β-estradiol (E2, E2758, Sigma-Aldrich, St. Louis, MO, USA), testosterone (T, 86500, Sigma-Aldrich, St. Louis, MO, USA), or RSG (E2408, Sigma-Aldrich, St. Louis, MO, USA). IL-6, E2, T, and RSG were dissolved in a solution containing 0.001% ethanol and 0.06% dimethyl sulfoxide (DMSO). The final concentrations added to the cell culture plates were 20 ng/mL for IL-6, 10 nM for E2, 10 μM for T, and 15 μM for RSG. The cells were incubated with these treatments for 24 h. An equivalent volume of ethanol + DMSO was added in each untreated control to a final concentration of 0.001% and 0.06%, respectively. For gene expression analysis, 3T3-L1 cells were harvested with Tripure^®^ isolation reagent from Roche Diagnostics (Basel, Switzerland), and RNA was isolated following the manufacturer’s instructions.

### 2.5. Gene Expression Analysis

Total RNA was isolated from 0.2 g of WAT and liver, respectively, using TriPure isolation reagent. RNA was quantified using the Take3 Microplate (BioTek, Winooski, VT, USA) set at 260 nm, and purity was assessed by the 260/280 ratio.

An amount of 1 µg of RNA was reverse transcribed to cDNA at 42 °C for 60 min with 25 U MuLV reverse transcriptase in 10 µL of retrotranscription reaction mixture containing 10 mM Tris-HCl (pH 9.0), 50 mM KCl, 0.1% Triton X-100, 2.5 mM ClMg_2_, 2.5 µM random hexamers, 10 U RNase inhibitor, and 500 µM each dNTP in a Gene Amp 9700 thermal cycler (Applied Biosystems, Lincoln, CA, USA). Each cDNA was diluted to 1/10. Real-time PCR (qPCR) was performed in WAT and liver for the genes listed in Table 1. The 18S ribosomal RNA gene and beta-actin were used as housekeeping genes in tissue and 3T3-L1 experiments. Information about the primers used is listed in Table 1. qPCR was performed using LightCycler 480 SYBR Green I Master technology on a LightCycler 480 System II rapid thermal cycler (Roche Diagnostics, Basel, Switzerland). Each reaction contained 5 µL of LightCycler 480 SYBR Green I Master (containing FastStart Taq DNA polymerase, dNTP mix, reaction buffer, MgCl_2_ and SYBR Green I dye), 0.5 µM of the forward and reverse specific primers, and 2.5 µL of the cDNA dilution in a final volume of 10 µL. Product specificity was confirmed in initial experiments by agarose gel electrophoresis and routinely by melting curve analysis.

### 2.6. Mitochondrial DNA Quantification

Total DNA was extracted from WAT and liver using Tripure^®^ (Roche Diagnostics, Basel, Switzerland) and quantified using a spectrophotometer set at 260 nm. The levels of mitochondrial DNA (mtDNA) were assessed by amplifying the mitochondrial gene NADH dehydrogenase subunit 4 and normalizing it to the nuclear-encoded gene 18S rRNA using qPCR. LightCycler^®^ 480 SYBR Green I Master Technology was used to perform the qPCR in a LightCycler^®^ 480 System II rapid thermal cycler from Roche Diagnostics. Each reaction contained 5 μL of LightCycler^®^ 480 SYBR Green I Master (containing FastStart Taq DNA polymerase, dNTP mix, reaction buffer, MgCl_2_ and SYBR Green I dye), forward and reverse primers (0.374 μM each), and 5 ng of the isolated DNA in a final volume of 10 µL. The oligonucleotide sequences used in real-time PCR are detailed in Table 1. Product specificity was confirmed in initial experiments by agarose gel electrophoresis and routinely by melting curve analysis.

### 2.7. Western-Blot Analyses

The effect of RSG on the protein levels of specific markers of insulin sensitivity (AKT, ADIPOR2, APPL1, AMPK and JNK) and mitochondrial function (PGC1α, PGC1β and TFAM) was measured by Western blot in WAT and liver. An amount of 25 µg of protein from WAT homogenates and 50 µg of protein from liver homogenates were separated by SDS-PAGE and transferred onto a nitrocellulose filter. Membranes were incubated in a blocking solution (5% non-fat powdered milk in phosphate-buffered saline, pH 7.5, containing 0.1% Tween^®^ 20, P1379, Sigma-Aldrich, St. Louis, MO, USA) for 1 h or overnight, depending on the primary antibody used. Primary antibodies against phospho- and total AKT (9271 and 9272, respectively), APPL1 (3858s), pAMPK (50081s), TFAM (7495), and phospho- and total JNK (9251s and 9255s, respectively) were purchased from Cell Signaling Technology (Danvers, MA, USA). AdipoR2 (sc-46755), total AMPK (sc-74461), and GAPDH (sc-25778) primary antibodies were from Santa Cruz Biotechnology (Santa Cruz, CA, USA). GAPDH was used as a loading control. Immunoblots were assessed using a commercial enhanced chemiluminescence kit (Bio-Rad, Hercules, CA, USA). Images were acquired with a ChemiDoc^TM^ XRS imaging system (Bio-Rad, Hercules, CA, USA) and analyzed using Quantity One© 1-D analysis software v. 4.6.5 (Bio-Rad, Hercules, CA, USA).

### 2.8. Statistical Analysis

For animal studies, 4–9 animals per group were utilized. All data are expressed as the mean ± standard error of the mean (SEM). For in vitro studies, data were obtained from at least three independent experiments performed in duplicate. Two-way ANOVA was used to determine statistically significant differences between groups in response to RSG (R) and sex (S) (*p* < 0.05) in WAT, in response to E2 and RSG, or T and RSG in 3T3-L1 adipocytes. The Fisher’s Least Significant Difference (LSD) test was used as a post hoc analysis (*p* < 0.05). All statistical analyses were performed using the statistical analysis software package IBM SPSS Statistics version 29.0.1.0 for Windows. The Ct values from the real-time PCR were analyzed using GenEx Standard Software v. 5.3.6 (MultiD Analyses, Göteborg, Sweden) and corrected for efficiency.

## 3. Results

### 3.1. RSG and Sex Effects on Body Weight and Adiposity

**Figure 1:**
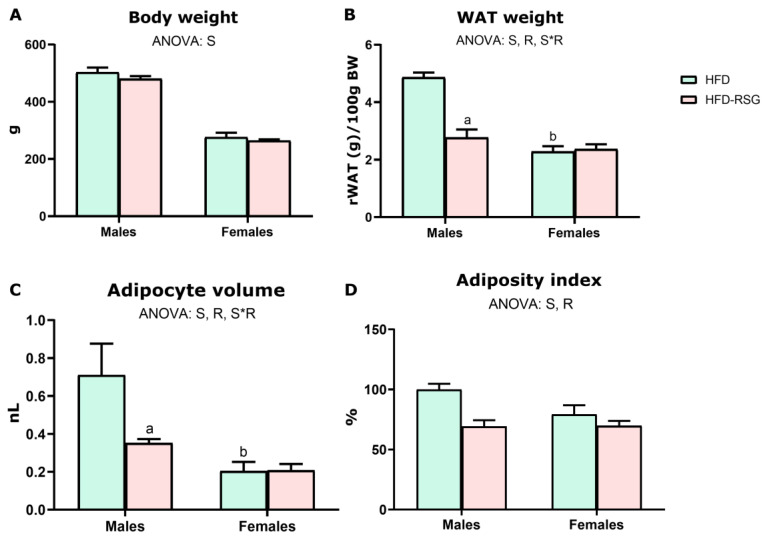
RSG and sex effects on body weight and adiposity. (**A**) Body weight. (**B**) White adipose tissue (WAT) weight refers to 100 g of body weight. (**C**) Adipocyte volume. (**D**) Adiposity index calculated as the sum of gonadal, retroperitoneal, and mesenteric depot weights relative to 100 g of body weight. Values are presented as the mean ± SEM (5–9 animals per group). Sex and RSG effects were analyzed by two-way ANOVA (*p* < 0.05): S—sex effect; R—RSG effect; and S*R—interactive effect. Fisher’s LSD post-hoc test (*p* < 0.05): (a) vs. HFD rats; (b) vs. male rats. WAT—white adipose tissue.

We aimed to investigate whether there is a sexual dimorphism in the mechanisms by which RSG enhances insulin sensitivity. Specifically, RSG enhances insulin sensitivity primarily by reducing adiposity and promoting fat storage, thereby preventing lipotoxicity. As expected, HFD-fed males showed higher body and WAT weights (Figure 1A,B), larger adipocytes (Figure 1C), and greater adiposity (Figure 1D) than females. RSG treatment did not alter body weight in either male or female HFD-fed rats (Figure 1A), although it led to a significant reduction in both relative WAT weight and adipocyte volume (Figure 1B,C), only in male rats. This reduction resulted in a significant decrease in the adiposity index, which was more noticeable in males than in females (31% vs. 12%, respectively) (Figure 1D). Consequently, RSG treatment mitigated the sexual dimorphism observed in WAT weight, adipocyte volume, and adiposity in HFD-fed animals.

### 3.2. RSG and Sex Effects on Glucose Tolerance and Serum Parameters

**Figure 2:**
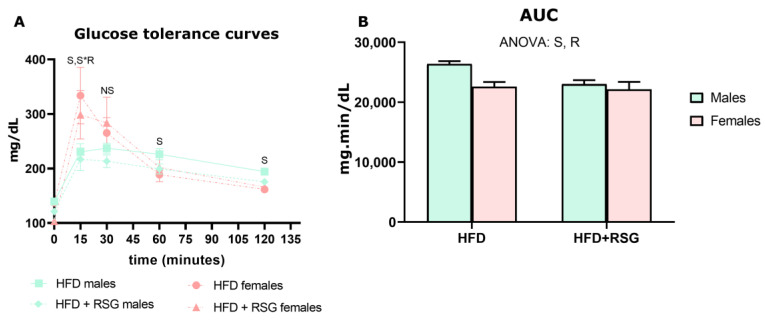
Glucose Tolerance Test. (**A**) Glucose tolerance curves. (**B**) AUC—area under the curve. Values are presented as the mean ± SEM (5–7 animals per group). Sex and RSG effects were analyzed by two-way ANOVA (*p* < 0.05): S—sex effect; R—RSG effect; and S*R—interactive effect. Fisher’s LSD post-hoc test (*p* < 0.05).

We measured markers of systemic insulin sensitivity in both male and female rats to determine if there was a sex-dependent effect in response to RSG. No sex differences were observed in the glucose serum levels of HFD-fed animals (Table 2). However, HFD males showed higher serum levels of insulin and leptin and lower levels of adiponectin compared to their female counterparts (Table 2). Consequently, the leptin/adiponectin ratio was nearly four times higher in males, indicating a heightened insulin resistance in this sex. Consistent with these findings, during the glucose tolerance test, untreated males exhibited sustained hyperglycemia at 60 and 120 min after glucose administration (Figure 2A), resulting in a larger area under the curve (AUC) compared to HFD females (Figure 2B). Collectively, these results suggested a poorer glycaemic control in HFD males accompanied by significant dyslipidemia, as evidenced by their higher serum levels of TG, NEFA, total cholesterol and LDL-c, and the lower HDL-c levels (Table 2). As a result, the higher LDL-c/HDL-c ratio in males suggested an increased cardiovascular risk.

As shown in Table 2 and Figure 2, the effect of RSG on improving insulin sensitivity was particularly pronounced in males. Although RSG significantly reduced serum glucose levels in both sexes, insulin levels decreased exclusively in HFD males, and no changes were observed in females. Additionally, RSG lowered the serum leptin levels in both sexes, with a more marked decrease observed in males (44% in males vs. 18% in females). Similarly, RSG had a notably greater impact on serum adiponectin levels in males compared to females (155% vs. 55%, respectively). As a result, RSG balanced adiponectin levels between sexes and decreased the leptin/adiponectin ratio by 78% in males compared to 61% in females, although it remained higher in males. These results indicate a more pronounced effect of RSG in male rats, which is in agreement with the lower AUC and the greater improvement in insulin sensitivity shown by this sex.

Additionally, RSG treatment effectively reduced TG levels in both sexes, resulting in a significant decrease in males compared to females. Similarly, NEFA levels decreased in males, with no significant effects observed in females. LDL-c levels were not affected by RSG, while HDL-c levels increased only in males. Consequently, the LDL-c/HDL-c ratio decreased more notably in males in response to RSG. Total cholesterol levels were unchanged by RSG treatment in either male or female rats.

### 3.3. RSG and Sex Effects on Insulin Sensitivity and Lipid Mobilization of WAT

**Figure 3:**
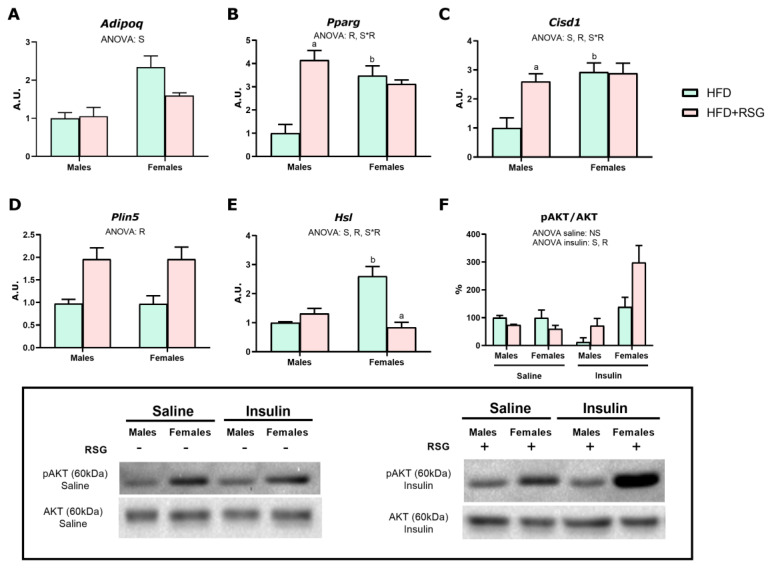
Insulin sensitivity and lipid mobilization in WAT. Gene expression analysis of (**A**) *Adipoq*, adiponectin; (**B**) *Pparg*—peroxisome proliferator-activated receptor gamma; (**C**) *Cisd1*—mitoNEET; (**D**) *Plin5*—perilipin 5; and (**E**) *Hsl*—lipase, hormone-sensitive. (**F**) pAKT/AKT ratio analyzed by Western blot. AKT serine-threonine kinase activation: insulin-treated animals received an intraperitoneal injection of insulin (5 U/kg) 20 min before sacrifice, while untreated animals (saline group) were injected with 0.9% sodium chloride solution (*w*/*v*). Values are presented as the mean ± SEM (4–7 animals per group). Sex and RSG effects were analyzed by two-way ANOVA (*p* < 0.05): S—sex effect; R—RSG effect; and S*R—interactive effect. Fisher’s LSD post-hoc test (*p* < 0.05): (a) vs. HFD rats; (b) vs. male rats.

We assessed the effects of sex and RSG on the expression levels of markers of insulin sensitivity and lipid mobilization in male and female rats. As shown in Figure 3A–C, HFD male rats showed lower expression levels of *Adipoq*, *Pparg,* and *Cisd1* (which encodes MitoNEET protein), suggesting that insulin sensitivity, adipogenesis and mitochondrial function may be reduced in this sex compared to females. Interestingly, RSG treatment attenuated the sex dimorphism observed in the *Adipoq* expression levels of HFD animals. (Figure 3A). As illustrated in Figure 3B,C, RSG induced a significant increase in the expression levels of both *Pparg* and *Cisd1* in male rats compared to females. These proteins improve insulin sensitivity by inducing adipogenesis and mitochondrial function. Hence, these results may account for the enhanced insulin sensitivity observed in RSG-treated males. In addition, RSG appears to equalize the levels of *Pparg* and *Cisd1* in both sexes, as no discernible effects of RSG were noted in females. Noteworthy is the fact that PPARγ is also known to stimulate adiponectin synthesis in WAT [27]. However, the decrease in adiponectin expression observed in the WAT of female rats in response to RSG does not align with the drug-induced increase in circulating adiponectin levels. This inconsistency might arise from adiponectin synthesis and secretion variations across different WAT depots. Notably, in response to RSG treatment, adiponectin expression levels in the periovarian WAT significantly increased in these animals (Supplementary data, Figure S1), explaining the elevated serum adiponectin levels observed in females under RSG treatment.

Regarding the markers of lipid mobilization, no differences were observed between sexes in *perilipin 5* mRNA levels (Figure 3D), in both HFD animals or RSG-treated animals. However, the expression levels of *Hsl* (Figure 3E), a marker of lipolysis, were significantly higher in HFD females, in agreement with the higher expression of adiponectin, *Pparg* and *Cisd1* observed in this sex. This finding aligns with the better insulin sensitivity profile observed in the WAT of HFD female rats. RSG treatment significantly increased perilipin 5 levels in both sexes and reduced *Hsl* expression exclusively in females (Figure 3D,E), both associated with improved insulin sensitivity. As shown in Figure 3F, insulin failed to induce AKT activation in HFD males, suggesting the presence of insulin resistance in these animals. Under basal conditions, RSG had no effect on AKT phosphorylation (Figure 3F). However, in female rats, RSG enhanced the response to insulin, suggesting a more pronounced effect of RSG in females (Figure 3F). These results suggest that the effects of RSG on the insulin signaling pathway were more pronounced in females.

### 3.4. RSG and Sex Effects on Mitochondrial Biogenesis and Dynamics in WAT

**Figure 4:**
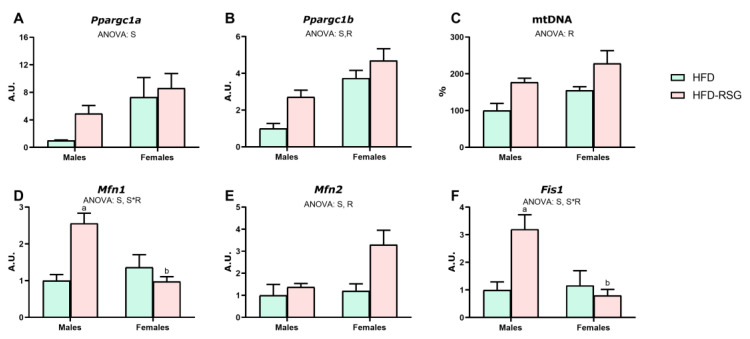
Mitochondrial biogenesis and dynamics in WAT. Gene expression analysis of (**A**) *Ppargc1a*—peroxisome proliferator-activated receptor gamma coactivator 1-alpha; (**B**) *Ppargc1b*—peroxisome proliferator-activated receptor gamma coactivator 1-beta; (**C**) mtDNA—mitochondrial DNA; (**D**) *Mfn1*—mitofusin 1; (**E**) *Mfn2*—mitofusin 2; (**F**) *Fis1*—fission, mitochondrial 1. Values are presented as the mean ± SEM (4–7 animals per group). Sex and RSG effects were analyzed by two-way ANOVA (*p* < 0.05): S—sex effect; R—RSG effect; and S*R—interactive effect. Fisher’s LSD post-hoc test (*p* < 0.05): (a) vs. HFD rats; (b) vs. male rats.

We analyzed the levels of mitochondrial biogenesis and dynamics to assess the effects of sex and RSG on WAT mitochondrial function. As depicted in Figure 4A–C, HFD males exhibited lower levels of *Ppargc1a*, *Ppargc1b* and mtDNA compared to females. However, the latter did not reach statistical significance (*p* = 0.059), which suggests reduced mitochondrial biogenesis in this sex. Regarding the expression levels of mitochondrial fusion (*Mfn1* and *Mfn2*) and fission (*Fis1*) markers, no sex differences were found in HFD rats (Figure 4D–F).

RSG treatment had no significant effect on *Ppargc1a* expression (Figure 4A) but induced an increase of both *Ppargc1b* (Figure 4B, 273% increase in males vs. 73% in females) and mtDNA (Figure 4C, 77% increase in males vs. 47% in females), attenuating the sex differences observed in HFD animals. Altogether, these results indicate that RSG treatment induced an enhancement of mitochondrial biogenesis, especially in males, which could contribute to the improvement of insulin sensitivity promoted by this drug in this sex. In contrast, a sex-dependent response to RSG was observed in the markers of mitochondrial dynamics, with male rats exhibiting greater gene expression of *Mfn1* and *Fis1*, whereas females showed higher expression of *Mfn2* (Figure 4D–F).

### 3.5. RSG and Sex Hormone Effects on Mitochondrial Biogenesis and Function in 3T3-L1 Adipocytes

**Figure 5:**
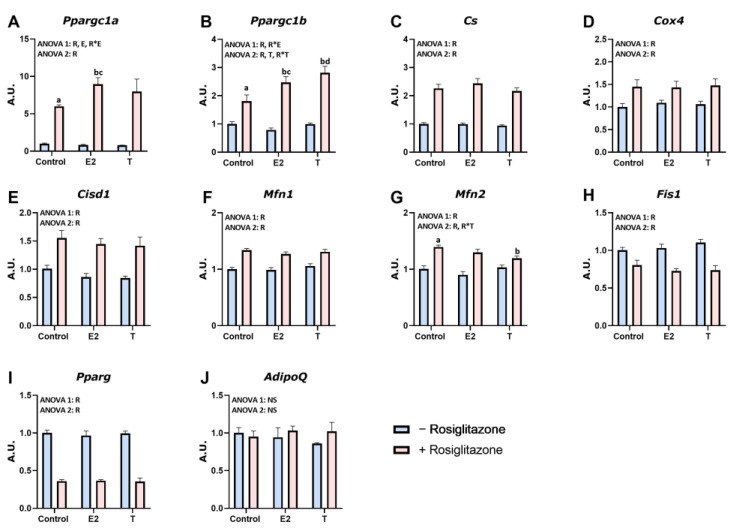
Mitochondrial biogenesis and dynamics in 3T3-L1 adipocytes. Adipocytes were treated for 24 h with IL−6 (20 ng/mL) combined with E2 (10 µM), T (10 µM), or RSG (15 µM), respectively. Gene expression analysis was measured of (**A**) *Ppargc1a*—peroxisome proliferator-activated receptor gamma coactivator 1-alpha; (**B**) *Ppargc1b*—peroxisome proliferator-activated receptor gamma coactivator 1-beta; (**C**) *Cs*—citrate synthase; (**D**) Cox4—cytochrome C oxidase subunit 4I1; (**E**) Cisd1—mitoNEET; (**F**) *Mfn1*—mitofusin 1; (**G**) *Mfn2*—mitofusin 2; (**H**) *Fis1*—fission, mitochondrial 1; (**I**) *Pparg*—peroxisome proliferator-activated receptor gamma; and (**J**) *Adipoq*—adiponectin. Values are presented as the mean ± SEM (n = 6). Differences between groups were analyzed by two-way ANOVA to detect a differential response to E2 and RSG (ANOVA1) and to T and RSG (ANOVA2) (*p* < 0.05), respectively: E—E2 effect, T—testosterone effect, E*R and T*R—interactive effect, and NS—non-significant. Fisher’s LSD post-hoc test (*p* < 0.05): (a) vs. control; (b) vs. control + RSG; (c) vs. E2; (d) vs. T.

Considering the sex-dependent response of WAT to RSG, we aimed to investigate whether the sex hormones E2 and T, in combination with RSG, affected 3T3-L1 adipocyte mitochondrial biogenesis. As shown in Figure 5A–J, the expression of the target genes analyzed did not show significant differences when treated with E2 or T alone. However, the combined treatment of RSG and E2 had an additive effect in two target genes, significantly upregulating *Ppargc1a* and *Ppargc1b* (Figure 5A,B) compared to RSG alone. Similarly, the combination of RSG and T enhanced the expression of *Ppargc1b* and decreased *Mfn2* expression.

RSG treatment induced an increase in the expression levels of *Ppargc1a*, *Ppargc1b*, *Cs*, *Cox4*, and *Cisd1*, confirming the results obtained in rats and indicating that RSG favors mitochondrial biogenesis and function in 3T3-L1 adipocytes (Figure 5A–E). Similarly, mitochondrial dynamics-related genes were regulated by RSG treatment, resulting in increased expression of *Mfn1* and *Mfn2* and decreased levels of *Fis1*, and suggesting enhanced mitochondrial fusion and reduced mitochondrial fission processes in response to RSG (Figure 5F–H). Surprisingly, RSG treatment reduced *Pparg* expression in 3T3-L1 adipocytes (Figure 5I), contrary to what happened in WAT and in agreement with previous studies where RSG reduces lipid content in mature adipocytes by reducing the expression of this gene. Therefore, the RSG effect on *Pparg* expression might be dependent on adipocyte maturity [32]. No effects on *Adipoq* mRNA levels were observed (Figure 5J).

### 3.6. RSG and Sex Effects on Inflammation, Hypoxia and Apoptosis in WAT

**Figure 6:**
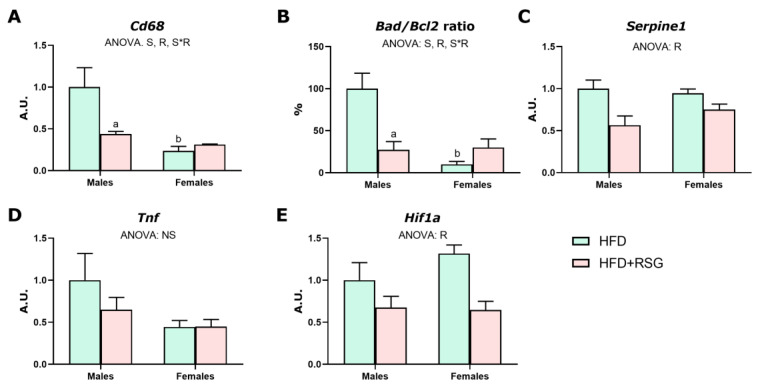
Markers of inflammation, hypoxia and apoptosis in WAT. Gene expression analysis of (**A**) *Cd68* antigen; (**B**) *Bad*/*Bcl2*—BCL2 associated agonist of cell death/BCL2 apoptosis regulator. (**C**) *Serpine1*, PAI-1—plasminogen activator inhibitor-1; (**D**) *Tnf*—tumor necrosis factor alpha; and (**E**) *Hif1a*—hypoxia inducible factor 1 alpha. Values are presented as the mean ± SEM (4–6 animals per group). Sex and RSG effects were analyzed by two-way ANOVA (*p* < 0.05): S—sex effect; R—RSG effect; and S*R—interactive effect. Fisher’s LSD post-hoc test (*p* < 0.05): (a) vs. HFD rats; (b) vs. male rats.

The ability of RSG to modify the expression of key markers of hypoxia and the low-grade chronic inflammation associated with HFD feeding was assessed. HFD males exhibited higher expression levels of the inflammation marker *Cd68* and an increased pro-apoptotic *Bad*/*Bcl2* ratio compared to females (Figure 6A,B). These findings are in accordance with the higher adiposity index and the more compromised function of WAT in male rats and, therefore, with their worse insulin sensitivity. No discernible differences between sexes were noted in *Serpine1*, *Tnf*, and *Hif1*a expression levels in HFD animals (Figure 6C–E).

RSG treatment notably ameliorated inflammation and decreased apoptosis, particularly in males, as evidenced by the reduction in the expression of *Cd68* and the ratio *Bad*/*Bcl2* (Figure 6A,B). Conversely, RSG treatment reduced the mRNA levels of *Serpine1* and *Hif1a* in both sexes (Figure 6C,E), and no effects were observed in *Tnf* expression (Figure 6D).

### 3.7. RSG and Sex Effects on Liver Weight and Lipid Content

During obesity, WAT dysfunction leads to ectopic accumulation of fat in the liver and peripheral tissues, thereby contributing to the development of insulin resistance. Since RSG also improves insulin sensitivity in the liver, we considered it important to assess whether this effect is also sex-dependent. Our results show that the hepatic lipid accumulation was more pronounced in HFD males than in females, evidenced by higher hepatic levels of TG and total cholesterol (Table 3). Notably, RSG treatment decreased lipid content in the liver of both sexes but more markedly in males, thereby attenuating sex differences.

### 3.8. RSG and Sex Effects on Adiponectin Signalling and Insulin Sensitivity in the Liver

**Figure 7:**
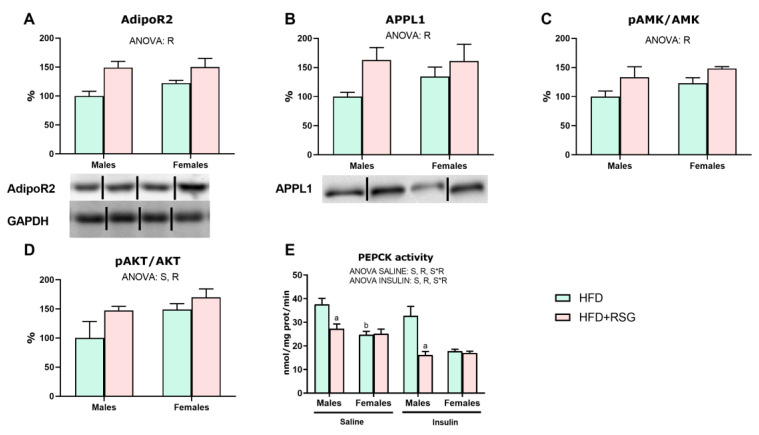
Markers of insulin sensitivity in the liver. Western blot analysis of (**A**) AdipoR2—adiponectin receptor 2; (**B**) APPL1—adaptor protein, phosphotyrosine interacting with PH domain and leucine zipper 1; (**C**) pAMPK/AMPK—phospho- and total protein kinase AMP-Activated Catalytic Subunit Alpha 1; (**D**) pAKT/AKT—pAKT/AKT, phospho- and total AKT serine-threonine kinase; and (**E**) PEPCK activity—phosphoenolpyruvate carboxykinase. To measure this enzymatic activity, insulin-treated animals received an intraperitoneal injection of insulin (5 U/kg) 20 min before sacrifice, while untreated animals (saline group) were injected with 0.9% sodium chloride solution (*w*/*v*). Values are presented as the mean ± SEM (5–7 animals per group). Sex and RSG effects were analyzed by two-way ANOVA (*p* < 0.05): S—sex effect; R—RSG effect; and S*R—interactive effect. Fisher’s LSD post-hoc test (*p* < 0.05): (a) vs. HFD rats; (b) vs. male rats.

To evaluate the effects of RSG on adiponectin function in the liver, we measured the expression levels of key components of its signaling pathway. In HFD animals, no sex differences were observed in the protein levels of AdipoR2 and APPL1, nor in the activation of AMPK (pAMPK/AMPK ratio) (Figure 7A–C). However, the pAKT/AKT ratio, a marker of activation of insulin signaling, was lower in HFD male rats compared to females (Figure 7D), suggesting a reduced insulin response in males. RSG treatment led to elevated levels of AdipoR2 and APPL1 proteins, as well as increased activation of AMPK and AKT in both sexes (Figure 7A–D). These results support the potential of this drug to enhance adiponectin signaling and improve insulin sensitivity in the liver.

To assess hepatic insulin resistance, the ability of insulin to suppress the activity of the gluconeogenic enzyme PEPCK was measured. As shown in Figure 7E, under basal conditions, PEPCK activity did not respond to insulin stimulation in HFD male rats, suggesting a marked insulin resistance in this sex. Conversely, as expected, PEPCK activity decreased in females in response to insulin, highlighting their better insulin sensitivity. RSG treatment decreased PEPCK activity only in males both in basal and insulin-stimulated conditions, with this decrease greater in the insulin-treated males. No effects of RSG were observed in females.

### 3.9. RSG and Sex Effects on Lipid Metabolism in Liver

**Figure 8:**
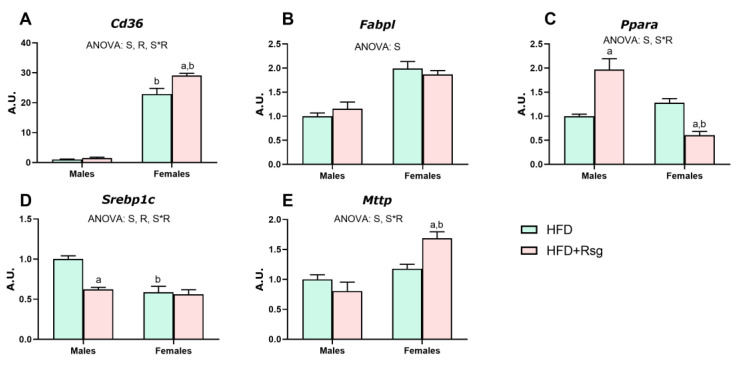
Lipid metabolism in the liver. Gene expression analysis of (**A**) *Cd36*—Cd36 antigen. (**B**) *Mttp*—microsomal triglyceride transfer protein. (**C**) *Srebp1c*—sterol regulatory element-binding protein 1. (**D**) *Ppara*—peroxisome proliferator-activated receptor alpha. (**E**) *Fabpl*—fatty acid binding protein 1, liver. Values are presented as the mean ± SEM (4–7 animals per group). Differences between sexes within groups and RSG effects were analyzed by two-way ANOVA (*p* < 0.05): S—sex effect; R—RSG effect; and S*R—interactive effect. Fisher’s LSD post-hoc test (*p* < 0.05): (a) vs. HFD rats; (b) vs. male rats.

One of the main benefits of the treatment with RSG is its ability to reduce lipid accumulation in the liver by modulating lipid metabolism. Compared to females, HFD males exhibited lower expression levels of key genes associated with fatty acid uptake (*Cd36*), transport (*Fabpl*) and oxidation (*Ppara*) (Figure 8A–C), accompanied by increased levels of *Srebp1c*, a master transcriptional regulator of lipogenesis (Figure 8D). RSG treatment did not alter lipid uptake and transport in males (Figure 8A,B,E), although it favored fatty acid oxidation (*Ppara)* and reduced fatty acid synthesis (*Srebp1c)* (Figure 8C,D). In contrast, in females, RSG treatment induced a significant increase in the expression levels of *Cd36* and *Mttp* (Figure 8A,E), the latter being crucial for assembling and secreting triglyceride-rich lipoproteins (VLDL), enhancing lipid export in this sex. Furthermore, a reduction in *Ppara* expression was specifically noted in females.

### 3.10. RSG and Sex Effects on Mitochondrial Biogenesis in Liver

**Figure 9:**
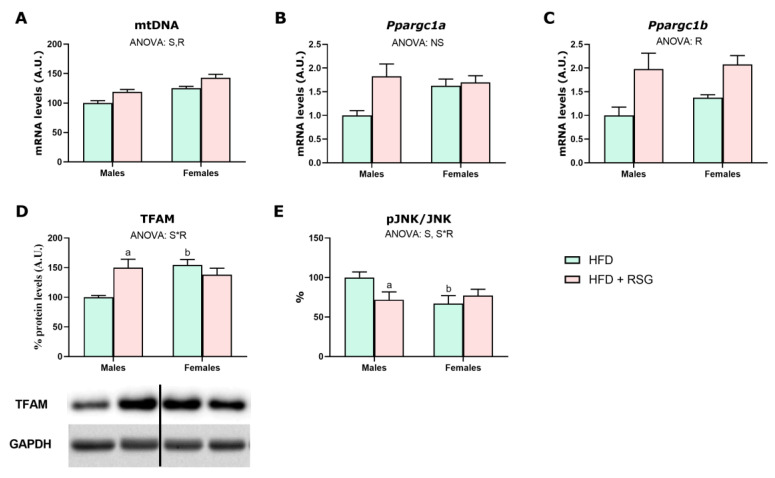
RSG and sex effects on hepatic mitochondrial biogenesis. (**A**) mtDNA—mitochondrial DNA, gene expression analysis of (**B**) *Ppargc1α*—peroxisome proliferator-activated receptor gamma coactivator 1-alpha and (**C**) *Ppargc1β*—peroxisome proliferator-activated receptor gamma coactivator 1-beta; and Western blot analysis of (**D**) TFAM protein levels; transcription factor A, mitochondrial; and (**E**) pJNK/JNK, phospho- and total c-Jun *N*-terminal kinase. Values are presented as the mean ± SEM (6–7 animals per group). Sex and RSG effects were analyzed by two-way ANOVA (*p* < 0.05): S—sex effect; R—RSG effect; and S*R—interactive effect. Fisher’s LSD post-hoc test (*p* < 0.05): (a) vs. HFD rats; (b) vs. male rats.

As shown in Figure 9, the liver of HFD males exhibited lower mtDNA levels and reduced TFAM protein levels compared to females, suggesting decreased mitochondrial biogenesis in males. Additionally, HFD males showed higher activation of JNK, a marker of oxidative stress and insulin resistance, as evidenced by their greater pJNK/JNK ratio [33]. RSG treatment enhanced mitochondrial biogenesis by increasing mtDNA and PGC1β levels in both sexes and TFAM protein levels in males. Notably, RSG reduced JNK activation in HFD males, while no effects were observed in females.

## 4. Discussion

This study aimed to determine whether a sexual dimorphism affects the efficacy of RSG in ameliorating WAT dysfunction within an HFD-induced obesity model. Our findings unveil a sex-dependent effect of RSG, with male rats undergoing greater insulin-sensitizing effects than female rats. Specifically, RSG-treated males exhibit a greater improvement in glucose tolerance accompanied by marked decreases in insulinemia, lipemia and LDL-c/HDL-c ratio due to increased HDL-c levels [34]. This differential response to RSG observed between male and female rats may be related to the different degrees of metabolic dysfunction induced by HFD. Notably, HFD males displayed a more pronounced metabolic imbalance compared to their female counterparts, with serum levels of insulin resistance markers such as insulin, TG, NEFA, leptin, and LDL-c accompanied by lower levels of insulin sensitivity markers such as adiponectin and HDL-c. Our findings indicate that RSG is particularly effective under conditions of heightened metabolic dysfunction, in agreement with previous studies showing improvements in glycemic and lipemic control in insulin-resistant animal models while having minimal impact on blood glucose levels in control animals [35,36]. Therefore, the greater insulin resistance observed in male rats under HFD conditions likely explains the different impact of RSG on this sex. In fact, RSG also improves systemic insulin sensitivity in females by elevating serum adiponectin and lowering glucose and TG levels, albeit to a lesser extent than in males. As a result, serum glucose levels reached the same values both in RSG-treated male and female rats, mitigating the sexual dimorphism observed in HFD animals.

HFD males showed higher adiposity and greater dysfunction in WAT compared to females. This increased adiposity, usually associated with hypertrophied and dysfunctional adipocytes, has been strongly correlated with the development of insulin resistance [4]. Notably, insulin administration failed to induce AKT activation in HFD males, confirming the lower insulin sensitivity in WAT of male rats [37]. In this context, hepatic fat accumulation was exacerbated in HFD-fed males, potentially due to the decreased ability of WAT to buffer fat as a result of its insulin resistance. Additionally, adiponectin signaling (AdipoR2, APPL1, and pAMPK/AMPK ratio) in the liver was found to be impaired in these animals, which could reduce fatty acid oxidation capacity, thereby increasing lipid accumulation and consequently exacerbating insulin resistance [38,39]. Conversely, in WAT of female rats, HFD induced an enhancement of lipogenic and adipogenic processes, as indicated by *Pparg* and *Cisd1* expression. These results suggest a better ability to safely store fatty acids in females and result in a lower degree of hepatic fat accumulation compared to males. Overall, these findings underscore the worse metabolic profile developed by male rats in response to HFD, which could be related to the more pronounced effects of RSG observed in this sex. Nevertheless, in WAT, RSG treatment led to greater AKT activation in females than in males and agrees with studies indicating that RSG enhances insulin sensitivity by improving insulin signaling [40]. Conversely, in males, RSG appears to improve insulin sensitivity by optimizing lipid handling. Previous studies have suggested that TZDs, including RSG, favor peripheral insulin action by promoting the redistribution of TG from the liver and muscle to WAT [41,42,43]. Our findings support this notion, as RSG treatment promotes lipid metabolism in the WAT of male rats by upregulating the expression levels of *Pparg* and *Cisd1*. The proteins codified by these genes, PPARγ and mitoNEET, decrease adipose tissue inflammation by inducing mitochondrial oxidative activity. Their upregulation, in males, correlates with a significant increase in *Plin5* levels, which are controlled by PPARγ and play a crucial role in enlarging lipid droplets by increasing TG content [44]. Perilipin 5 achieves this by creating a barrier that restricts the access of soluble lipases to stored lipids, thereby preventing TG hydrolysis [45]. This increase in *Plin5* levels was also seen in females in response to RSG, although it did not correlate with higher *Pparg* levels. Thus, RSG would improve insulin sensitivity in males by promoting adipogenesis *(Pparg)*, thereby favoring healthy fat storage *(Plin5)* and contributing to reducing WAT inflammation [6]. Indeed, RSG treatment decreased inflammation *(Tnf)*, macrophage infiltration *(Cd68)*, and apoptosis *(Bad*/*Bcl2)* only in males. The reduction of WAT inflammation exhibited by these animals occurs in parallel to the decrease of serum leptin levels and correlates with the lower WAT weight observed in these animals. Of note, leptin is recognized for its chemotactic properties that promote inflammation [46,47]. Interestingly, the increased adipogenesis induced by RSG in male rats leads to healthier fat storage without increasing WAT weight and adiposity. This can be explained by RSG promotion of hepatic fat oxidation *(Ppara)* as well as reduction of fatty acid synthesis *(Srebp1c)* in males. In contrast, in females, RSG reduces fat oxidation *(Ppara)* while enhancing hepatic lipid export *(Cd36*, *Mttp)*. Through these mechanisms, RSG promotes lipid oxidation and redistribution, reducing hepatic fat accumulation and improving insulin sensitivity in both sexes.

In obesity, as adipocytes expand and become metabolically dysregulated, their mitochondrial function undergoes significant impairment. Notably, optimal mitochondrial function is crucial for supplying energy to the biosynthetic processes undertaken by WAT, including adipokine production [48]. Dysfunctional adipocytes exhibit disrupted mitochondrial dynamics and oxidative capacity, reduced ATP production, and increased generation of reactive oxygen species, thereby exacerbating cellular stress and inflammation [49,50,51]. In the present study, we observed a significant sexual dimorphism in the WAT mitochondrial function, with HFD-fed males showing impaired mitochondrial biogenesis compared to females, based on their lower *Ppargc1b* expression and the lesser mtDNA content (*p* = 0.059). These findings agree with the lower circulating adiponectin levels and reduced WAT expression observed in males, suggesting a more severe degree of insulin resistance in this sex and highlighting males as more susceptible to the detrimental effects of HFD. Notably, one of the mechanisms used by RSG to improve energy metabolism in WAT is to induce mitochondrial function through the regulation of *Ppargc1b* expression levels [52], as observed in both male and female rats.

Additionally, RSG promoted mitochondrial proliferation, as denoted by the increase in mtDNA levels. These findings align with the higher expression of mitochondrial function markers (*Ppargc1a*, *Ppargc1b*, *mtDNA*, *Cox4*, *Cs*, *Cisd1*, *Mfn1*, *Mfn2*, and *Fis1*) in 3T3-L1 adipocytes treated with RSG. Notably, while treatment with E2 or T alone did not affect the target genes studied, their combination with RSG enhanced the drug’s effects on *Ppargc1a* and *Ppargc1b* expression. Thus, sex hormones may partially modulate RSG’s effects on mitochondrial biogenesis. Mitochondrial dynamics in WAT were also regulated by RSG in a sex-dependent manner, with males showing an increase in *Mfn1* and *Fis1* levels, while females exhibited higher levels of *Mfn2*, which, apart from its role in mitochondrial fusion, is involved in the maintenance of a correct mitochondrial oxidative capacity [53]. Therefore, RSG enhances both mitochondrial fusion and fission in male rats, improving the quality control system that eliminates dysfunctional mitochondria [53,54,55]. The balanced combination of these two processes ensures the efficient removal of damaged mitochondria, sustaining a better energy supply, reducing oxidative stress, and enhancing WAT insulin sensitivity in males.

Notably, under HFD conditions, males developed greater adiposity and hepatic steatosis compared to females, along with worse hepatic mitochondrial function and higher activation of stress-activates protein kinase JNK (pJNK/JNK). However, RSG enhanced mitochondrial proliferation (mtDNA) and biogenesis (TFAM) and reduced activation of JNK (pJNK/JNK), with these effects being more pronounced in males than in females. The significant improvement in hepatic insulin sensitivity observed in males may be attributed to the enhanced adiponectin signaling observed in this tissue. RSG treatment led to elevated protein levels of AdipoR2 and APPL1 and activated AMPK in both sexes, with a particularly pronounced effect in males. Consistently, RSG increased PPARα expression levels exclusively in males. As a result, both AMPK and PPARα, as key mediators of the adiponectin signaling pathway, could contribute to the improvement of liver insulin sensitivity in response to RSG by stimulating mitochondrial function and increasing FA oxidation [39]. Our data show that RSG increased mtDNA content and the levels of *Pgc1b* and TFAM, especially in males. These changes contributed to reducing hepatic steatosis and improving insulin sensitivity in this sex, as evidenced by the lower Srebp1c expression, the greater AKT activation, and the higher insulin-induced suppression of PEPCK activity observed compared to females. In contrast, in female rats, RSG also improved insulin sensitivity, although the effects were more discreet and reduced intrahepatic lipid accumulation by promoting the maturation and secretion of pre-VLDL *(Mttp*, *Cd36)*, consistent with previous reports [56]. Overall, our results illustrate the sex-dependent effects of RSG, with male rats exhibiting a more pronounced response to the treatment, likely due to their heightened degree of insulin resistance in response to HFD.

## 5. Conclusions

Our study underscores the relevance of sex differences in the anti-diabetic effects of RSG in WAT and liver, which seems to be associated with the severity of metabolic dysfunction. We found that RSG treatment markedly counteracts the greater degree of obesity, insulin resistance, inflammation, lipid dysregulation, and WAT mitochondrial dysfunction induced by HFD, with these effects being significantly more pronounced in male rats. RSG promotes healthier fat storage in adipose tissue, reduces lipemia, and enhances insulin sensitivity, particularly in males. Additionally, RSG improves hepatic insulin sensitivity by decreasing ectopic fat deposition and promoting fat oxidation in the liver. These findings emphasize the importance of considering sex-specific response patterns in the development of therapeutic strategies for metabolic disorders, paving the way for more effective and tailored treatments.

## Figures and Tables

**Table 1:** Oligonucleotide primer sequences used in real-time PCR amplification and product length.

Gene	Forward (5′ → 3′) Reverse (3′ → 5′)	Accession Number	Product Length (pb)
*r18S*	CGA ACC TCC GAC TTT CGT TCT GCG GTG AAA TTC TTG GAC CGG	NR_046237.1	90
*mActb*	CCG GGA CCT GAC GGA CTA CCT CAT GAA GAT AAT AGT GAT GAC TTG GCC GTC AGG CAG CTC	NM_007393.5	205
*rAdipoQ*	GAA GGG AGA GAA GGG AGA CG CGC TGA ATG CTG AGT GAT ACA	NM_144744.3	158
*mAdipoQ*	GTT GCA AGC TCT CCT GTT CC TCT CCA GGA GTG CCA TCT CT	NM_009605.5	192
*rBad*	AGA GTT TGA GCC GAG TGA GC ACT CCG GGT CTC CAT AGT CC	NM_022698.1	186
*rBcl2*	CTT CTT TGA GTT CGG TGG GGT GGA GAA ATC AAA CAG AGG TCG C	NM_016993.1	151
*rCd36*	CTCACACAACTCAGATACTGCTG TCCAAACACAGCCAGGACAG	NM_031561	200
*rCd68*	CCC GAA CAA AAC CAA GGT CC CTG CGC TGA GAA TGT CCA CT	NM_001031638.1	195
*rCisd1*	ACG CTA AAG AGA GTC GCA CC CAT CGC AGA ACG GGA ACT TTT	NM_001106385.2	150
*mCisd1*	GCT GTG CGA GTT GAG TGG AT TGG TGC GAT TCT CTT TAG CGT A	NM_134007.4	103
*mCox4*	AGA AGG CGC TGA AGG AGA AGG A CCA GCA TGC CGA GGG AGT GA	NM_009941.3	386
*mCs*	GTT AGC TGG AGA CGC TTT AGA GGC CTG GAA GGA AAC	NM_026444.4	158
*rFabpl1*	TGC GAA CTG GAG ACC ATG AC TGT AGA CGA TGT CAC CCA GTG	NM_012556	157
*rFis1*	TGT AGC GTG AAG GAT TGC AG CTT CAT CTC TGG GCA TCC AT	NM_001105919.1	197
*mFis1*	CTG GCC GTG GGC AAC TAC CAG CCC TCG CAC ATA CTT TAG A	NM_001347504.1	63
*rHif1a*	CCC CTT CCT CCT TCA TTT TC GGA CAA ACT CCC TCA CCA AA	NM_024359.1	159
*rHsl*	GGA CAG TGA TCC CAG GAA CG ATG CTG TGT GAG AAT GCC GA	NM_012859.1	151
*Mt-nd1*	TAC ACG ATG AGG CAA CCA AA GGT AGG GGG TGT GTT GTG AG	NC_001665	162
*rMfn1*	GAC GAC AGC ACA TGG AAA GA CTT GCC TGA AAT CCT TCT GC	NM_138976.1	142
*mMfn1*	CAA CAC TGA TGA ACA CGG AGA AA CCC AAC GGT TAT TCA GAA TGA AG	NM_024200.5	90
*rMfn2*	AGG AAA TTG CTG CCA TGA AC GTC TCT TCT CGG TGC AGG TC	NM_130894.4	174
*mMfn2*	TGC TGG TGG CCA ACT CAG A GGA GAG ACG TTC ACT CAC TTT GTG	NM_001355590.1	77
*rMttp*	ACCTGCGAACCTGTCCAACG CCAGGATGGCTTCCAGTGAG	NM_001107727	182
*rPlin5*	CAC TGT GCT GAG GCG CT ACG CAC AAA GTA GCC CTG TT	NM_001134637.1	181
*rPpargc1a*	ATC TAC TGC CTG GGG ACC TT ATG TGT CGC CTT CTT GCT CT	NM_031347	180
*mPpargc1a*	AAC CAC ACC CAC AGG ATC AGA CTC TTC GCT TTA TTG CTC CAT GA	NM_008904.3	74
*rmPpargc1b*	ACT ATG ATC CCA CGT CTG AAG AGT C CCT TGT CTG AGG TAT TGA GGT ATT C	NM_176075	152
*rPpara*	TGCCTTCCCTGTGAACTGAC GCTTCAAGTGGGGAGAGAGG	NM_013196	151
*rPparg*	TCA GAG GGA CAA GGA TTC ATG A CAC CAA AGG GCT TCC GCA GGC T	NM_013124	61
*mPparg*	TTT TCA AGG GTG CCA GTT TC AAT CCT TGG CCC TCT GAG AT	NM_011146.4	198
*rSerpine1*	GAC AAT GGA AGA GCA ACA TG ACC TCG ATC TTG ACC TTT TG	NM_012620.3	205
*rSrebp1c*	CGCTACCGTTCCTCTATCAATGAC AGTTTCTGGTTGCTGTGCTGTAAG	NM_001276707	140
*rTnf*	GGT TCC GTC CCT CTC ATA CA AGA CAC CGC CTG GAG TTC T	NM_012675.3	132

*r*—rat; *m*—mouse.

**Table 2:** Serum parameters.

		HFD			HFD + RSG			ANOVA
**Glucose (mg/dL)**	Males	156	±	2.3	144	±	5.4	R
	Females	154	±	5	136	±	5.1	
**Insulin (** **µg/** **L)**	Males	0.85	±	0.10	0.61	±	0.04 ^a^	S, R, S*R
	Females	0.50	±	0.01 ^b^	0.52	±	0.03 ^b^	
**Adiponectin (** **µ** **g/mL)**	Males	21.3	±	1.6	54.3	±	3.0 ^a^	S, R, S*R
	Females	36.6	±	0.6 ^b^	56.7	±	4.3 ^a^	
**Leptin (ng/mL)**	Males	23.3	±	3.2	13.0	±	2.1	S, R
	Females	7.70	±	1.04	6.29	±	0.38	
**Leptin/Adiponectin**	Males	1.07	±	0.16	0.24	±	0.04 ^a^	S, R, S*R
	Females	0.28	±	0.02 ^b^	0.11	±	0.021 ^ab^	
**TG (mg/dL)**	Males	314	±	12	163	±	4 ^a^	S, R, S*R
	Females	229	±	17 ^b^	166	±	4 ^a^	
**NEFA (µg/L)**	Males	0.73	±	0.01	0.57	±	0.01 ^a^	R, S*R
	Females	0.60	±	0.03 ^b^	0.59	±	0.06	
**Total cholesterol (mM)**	Males	2.67	±	0.13	2.55	±	0.05	S
	Females	1.93	±	0.08	2.02	±	0.08	
**LDL-c (mM)**	Males	0.52	±	0.06	0.45	±	0.03	S
	Females	0.34	±	0.03	0.29	±	0.04	
**HDL-c (mM)**	Males	0.44	±	0.07	1.08	±	0.05 ^a^	R, S*R
	Females	0.76	±	0.05 ^b^	0.89	±	0.10	
**LDL-c/HDL-c**	Males	1.20	±	0.26	0.42	±	0.03	S, R
	Females	0.55	±	0.11	0.30	±	0.04	

TG—triglycerides; NEFA—non-esterified fatty acids; LDL-c—low-density lipoprotein cholesterol; HDL-c—high-density lipoprotein cholesterol; and LDL-c/HDL-c ratio. Values are presented as the mean ± SEM (6 animals per group). Sex and RSG effects were analyzed by two-way ANOVA (*p* < 0.05): S—sex effect; R—RSG effect; and S*R—interactive effect. Fisher’s LSD post-hoc test (*p* < 0.05): (**^a^**) vs. HFD rats; (**^b^**) vs. male rats.

**Table 3:** RSG and sex effects on liver weight and hepatic lipid content.

		HFD			HFD + RSG			ANOVA
Hepatic specific weight (g/100 g BW)	Males	2.83	±	0.05	2.69	±	0.06	S
	Females	2.54	±	0.07	2.47	±	0.06	
TG (mg/g tissue)	Males	49.8	±	3.2	19.8	±	1.2 ^a^	S, R, S*R
	Females	25.3	±	0.6 ^b^	19.5	±	1.9 ^a^	
Total cholesterol (mg/g tissue)	Males	13.9	±	1.3	7.49	±	1.19 ^a^	S, R, S*R
	Females	7.38	±	0.15 ^b^	6.42	±	0.23 ^a^	

Values are presented as the mean ± SEM (5–7 animals per group). Sex and RSG effects were analyzed by two-way ANOVA (*p* < 0.05): S—sex effect; R—RSG effect; and S*R—interactive effect. Fisher’s LSD post-hoc test (*p* < 0.05): ^a^, vs. HFD rats; ^b^, vs. male rats. TG—triglycerides.

## Data Availability

The original contributions presented in the study are included in the article/Supplementary Material, further inquiries can be directed to the corresponding author.

## Supplementary Materials

The following supporting information can be downloaded at: https://www.mdpi.com/article/10.3390/nu16183063/s1, Figure S1. Adiponectin expression levels in gonadal WAT. gWAT, gonadal white adipose tissue; HFD, high fat diet; HFD + Rsg, high fat diet treated with rosiglitazone (100mg/Kg diet). Values are expressed as the mean ± SEM of 7 or 8 animals per group. ANOVA (*p* < 0.05): S, sex effect; and R, rosiglitazone effect. Fisher’s LSD post-hoc test (*p* < 0.05): a, female vs male; b, HFD + Rsg vs. HFD.
