# Supplementary material for: Impact of Sex on the Therapeutic Efficacy of Rosiglitazone in Modulating White Adipose Tissue Function and Insulin Sensitivity
Source: Nutrients. 2024 Sep 11;16(18):3063. doi: 10.3390/nu16183063 (PMC11434741; doi:10.3390/nu16183063)
Supplement: Supplementary file 1 [file nutrients-16-03063-s001.zip › nutrients-3161589-supplementary.pdf]

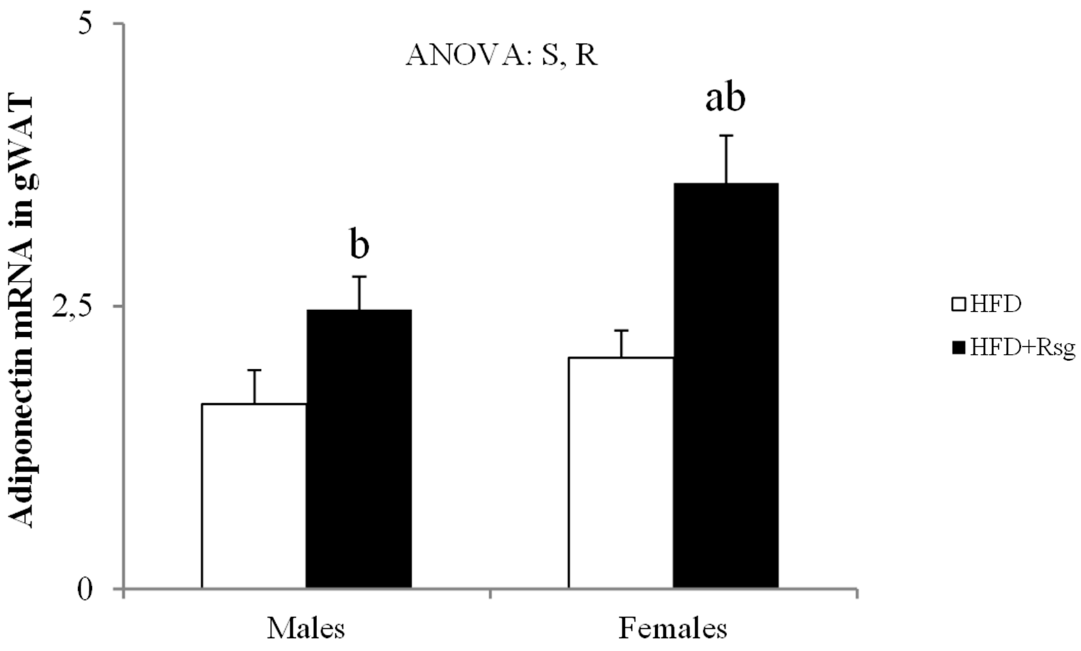

Supplementary data. Figure S1. Adiponectin expression levels in gonadal WAT. gWAT, gonadal white adipose tissue; HFD, high fat diet; HFD+Rsg, high fat diet treated with rosiglitazone (100mg/Kg diet). Values are expressed as the mean ± SEM of 7 or 8 animals per group. ANOVA (p<0.05): S, sex effect; and R, rosiglitazone effect. Fisher's LSD post-hoc test ($p < 0.05$): a, female vs male; b, HFD+Rsg vs HFD.
